# Aluminium-Based Metal–Organic Framework Nano Cuboids and Nanoflakes with Embedded Gold Nanoparticles for Carbon Dioxide Fixation with Epoxides into Cyclic Esters

**DOI:** 10.3390/ijms25021020

**Published:** 2024-01-13

**Authors:** Gabriela Kopacka, Kinga Wasiluk, Pawel W. Majewski, Michał Kopyt, Piotr Kwiatkowski, Elżbieta Megiel

**Affiliations:** 1 Faculty of Chemistry, University of Warsaw, Pasteur 1, 02-093 Warsaw, Poland; g.kopacka@student.uw.edu.pl (G.K.); k.wasiluk3@student.uw.edu.pl (K.W.); pmajewski@chem.uw.edu.pl (P.W.M.); m.kopyt2@student.uw.edu.pl (M.K.); pkwiat@chem.uw.edu.pl (P.K.); 2 Biological and Chemical Research Centre, University of Warsaw, Żwirki i Wigury 101, 02-089 Warsaw, Poland

**Keywords:** metal–organic frameworks, cyclic carbonates, heterogeneous catalysts, gold nanoparticles, CO_2_ fixation, epoxides

## Abstract

The fixation of carbon dioxide with epoxides is one of the most attractive methods for the green utilisation of this greenhouse gas and leads to many valuable chemicals. This process is characterised by 100% atom efficiency; however, an efficient catalyst is required to achieve satisfactory yields. Metal–organic frameworks (MOFs) are recognised as being extremely promising for this purpose. Nevertheless, many of the proposed catalysts are based on ions of rare elements or elements not entirely safe for the environment; this is notable with commercially unavailable ligands. In an effort to develop novel catalysts for CO_2_ fixation on an industrial scale, we propose novel MOFs, which consist of aluminium ions coordinated with commercially available 1,4-naphthalene dicarboxylic acid (Al@NDC) and their nanocomposites with gold nanoparticles entrapped inside their structure (AlAu@NDC). Due to the application of 4-amino triazole and 5-amino tetrazole as crystallization mediators, the morphology of the synthesised materials can be modified. The introduction of gold nanoparticles (AuNPs) into the structure of the synthesised Al-based MOFs causes the change in morphology from nano cuboids to nanoflakes, simultaneously decreasing their porosity. However, the homogeneity of the nanostructures in the system is preserved. All synthesised MOF materials are highly crystalline, and the simulation of PXRD patterns suggests the same tetragonal crystallographic system for all fabricated nanomaterials. The fabricated materials are proven to be highly efficient catalysts for carbon dioxide cycloaddition with a series of model epoxides: epichlorohydrin; glycidol; styrene oxide; and propylene oxide. Applying the synthesised catalysts enables the reactions to be performed under mild conditions (90 °C; 1 MPa CO_2_) within a short time and with high conversion and yield (90% conversion of glycidol towards glycerol carbonate with 89% product yield within 2 h). The developed nanocatalysts can be easily separated from the reaction mixture and reused several times (both conversion and yield do not change after five cycles). The excellent performance of the fabricated catalytic materials might be explained by their high microporosity (from 421 m^2^ g^−1^ to 735 m^2^ g^−1^); many catalytic centres in the structure exhibit Lewis acids’ behaviour, increased capacity for CO_2_ adsorption, and high stability. The presence of AuNPs in the synthesised nanocatalysts (0.8% *w*/*w*) enables the reaction to be performed with a higher yield within a shorter time; this is especially important for less-active epoxides such as propylene oxide (two times higher yield was obtained using a nanocomposite, in comparison with Al-MOF without nanoparticles).

## 1. Introduction

The increase in CO_2_ emission from anthropogenic sources is one of the crucial reasons for the climate change observed throughout the last few decades. Global warming, related to the increasing concentration of greenhouse gases in the atmosphere, is responsible for decreasing glaciers’ surface, increasing desert areas, rising ocean levels, climate anomalies, and many other alarming phenomena [1]. Carbon dioxide from anthropogenic sources has the most considerable contribution among greenhouse gases. In 2022, it was calculated that the combustion of fossil fuels released 36.1 ± 0.3 Gt of CO_2_, which accounted for 70% of its global emission into the atmosphere [1,2].

Carbon capture and utilization (CCU) is recognised as the most recommended strategy for decreasing anthropogenic CO_2_ emission [3]. While capture from exhaust gases and storing methods have been well developed, the utilisation of CO_2_ for useful products is still a challenge [4], due primarily to the high thermodynamic and kinetic stability of this compound [5]. To activate CO_2_, reactive substrates such as hydrogen, amines, or epoxides should be used, which decrease the thermodynamic limitation; meanwhile, an effective catalyst is required to lower the kinetic barrier [6].

**Scheme 1:**
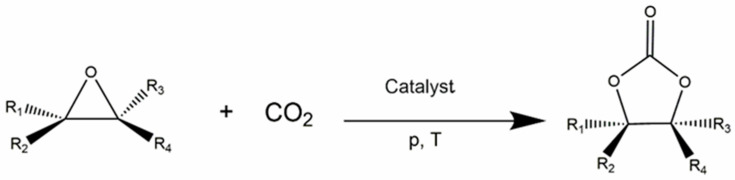
Scheme of cycloaddition of CO_2_ to epoxides.

The fixation of CO_2_ using epoxides leading to cyclic esters, also called cycloaddition of CO_2_, runs with 100% atom efficiency, meaning all substrates’ atoms are built into the product molecule (Scheme 1).

Cyclic organic esters (COEs) are a broad group of compounds with many applications; among others, they can be used as electrolytes in lithium-ion batteries, polar high-boiling-point solvents, components of cosmetics, and monomers for the production of polymers (polycarbonates, polyurethanes); their present world annual production is ca. 600 thousand tons [5]. Most often, COEs are liquids with high dielectric constants; they are non-toxic, soluble in water, biocompatible, and biodegradable into safe compounds, and they are recognised as green solvents. Currently, COEs are produced using technologies based on highly toxic phosgene [2]. Implementing the reaction cycloaddition CO_2_ to epoxides into new technologies of COEs production is an attractive alternative based on sustainable development rules [7].

The addition of CO_2_ to epoxides requires the use of a catalyst that can be employed in a homogeneous or heterogeneous system [8,9,10,11], whereas the last, although usually gives a lower yield, enables easy separation and reuse of the catalyst. Thus, heterogeneous catalysts are especially desired. Among heterogeneous catalysts, metal–organic framework materials (MOFs) are up and coming for the fixation of CO_2_ [12,13,14,15]. MOFs are crystalline and porous nanostructures in which metal ions are coordinated by organic molecules (bifunctional ligands) [16,17]. Importantly, MOFs with an adequately designed morphology and composition can adsorb CO_2_ inside the pores and contain active catalytic centres with Lewis acid behaviour due to the presence of not fully coordinated metal ions [18,19,20]. Usually, MOFs are employed in the cycloaddition of CO_2_ to epoxides with co-catalysts, providing the nucleophilic moieties necessary to form the halo-alkoxy transition form between CO_2_ and epoxide molecules. Most often, tetramethylammonium bromide (TBAB) plays this role [13,21].

Recently, a few reports revealed that the composite materials built of MOFs with catalytically active substances whose molecules are entrapped inside their pores can be more effective than their application separately [7,22,23,24]. In 2018, Ding and Jiang showed that the incorporation of poly(ionic liquid) built of imidazole moieties inside of MOF structure (chromium ions coordinated by terephthalic acid ligand MIL-101) gives heterogeneous effective and selective catalyst for the cycloaddition CO_2_ to epoxides [25]. However, the preparation of this material is rather complicated and consists of a multistep procedure using expensive chemicals, making it difficult to employ on an industrial scale. Furthermore, the high toxicity of chromium(III) ions causes the production and application of this type of MOF material to pose a risk to the environment.

Baumick and co-workers [26] proved that the cerium(III) based MOF with 1,4-naphthalene dicarboxylic acid (1,4-NDC) as a linker is an efficient catalyst for the cycloaddition of CO_2_ to epoxides. The authors also showed that 4-amino triazole or 5-amino tetrazole during the MOF crystallisation causes the formation of a more ordered structure during the MOF crystallisation whilst not being incorporated into the MOF’s network (crystallisation mediators). The fabricated Ce-MOF works well as a heterogeneous catalyst for CO_2_ cycloaddition to several epoxides. Nonetheless, cerium is a rare element, and its concentration in Earth’s crust is only 0.0004%; thus, applying such a catalyst on a larger scale would be costly.

Very recently, novel cobalt-based MOFs with abundant O/N species were reported to be highly effective at catalysing the cycloaddition of CO_2_ to epichlorohydrin as a model epoxide [27]. A specially designed ligand that is not commercially available was used to synthesise this material. It is worth pointing out that materials with cobalt must be carefully used on a larger scale because of the toxicity of this heavy metal.

So far, only a few papers report the successful application of nanostructural gold for the fixation of CO_2_. At the same time, gold nanoparticles (AuNPs) and other noble metal nanoparticles are extensively studied because of their unique properties and exceptional effectivity in the heterogeneous catalysis field [28,29,30,31,32,33]. In 2021, Yang et al. [34] proved that gold nanoclusters (AuNPs with diameter < 2 nm) could catalyse the cycloaddition of CO_2_ to propylene oxide using dimethyl aminopyridine (DMAP) as a co-catalyst. Importantly, they also showed weak adsorption of CO_2_ onto the gold surface, which may explain the low yields obtained in the cycloaddition [28]. Based on these insights, we hypothesised that immobilising nanostructural gold into the MOF with a high capacity to adsorb CO_2_ should improve its catalytic performance.

Herein, we report the synthesis of novel highly porous aluminium-based MOFs with 1,4-naphthalene dicarboxylic acid as a linker and their composites with nanostructural gold. The successful application of all these materials in the cycloaddition of CO_2_ to a series of model epoxides (glycidol, epichlorohydrin, styrene oxide, propylene oxide) is also presented. We employed 4-amino triazole and 5-amino tetrazole in the materials synthesis as mediators of crystallisation, and two types of Al-MOFs have been obtained differing slightly in morphology labelled as Al4@NDC, Al5@NDC, respectively. So far, Al-based MOFs with NDC as a linker have been obtained using the solvothermal method in water solutions [35,36,37].

The composite materials (AlAu@NDC) were fabricated through the “ship in a bottle” strategy using ultra-small gold nanoparticles AuNPs (with a diameter of ca. 3 nm), which were introduced during the MOFs’ crystallisation processes.

The fabricated Al-MOFs and their composites with AuNPs show good and excellent results in the cycloaddition of carbon dioxide to tested model epoxides. They seem perfect candidates for catalysts in the sustainable fixation of CO_2_ on an industrial scale.

## 2. Results and Discussion

**Figure 1:**
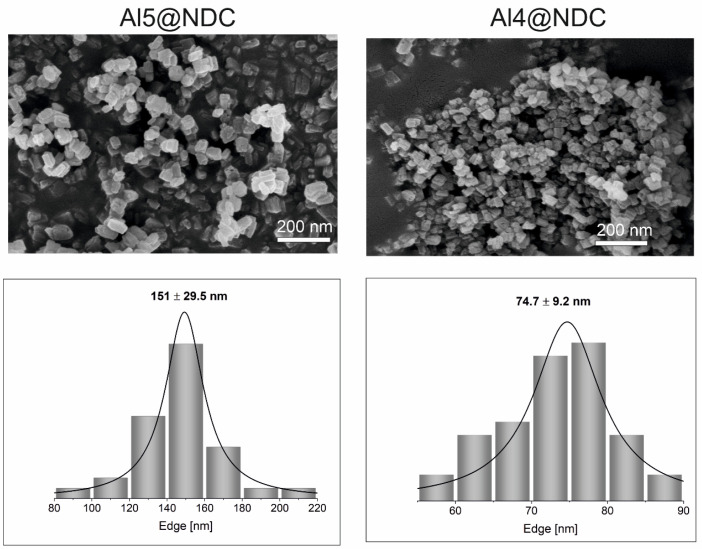
SEM images and histograms determined on the base of these images for Al-MOF nanocubes obtained using 5-amino tetrazole (Al5@NDC) and 4-amino triazole (Al4@NDC) as crystallisation mediators.

The Al-MOFs were synthesised using 1,4-naphthalene dicarboxylic acid as the organic linker and 4-aminotriazole (4-Atz) or 5-amino tetrazole (5-Atrz) as mediators of crystallisation. Baumick et al. [26] employed this linker and mediators to prepare the cerium-based MOFs. In this case, only one type of material for both mediators was obtained. However, we observed significant differences in the morphology of the materials obtained using 4-aminotriazole and 5-aminotetrazole (Al4@NDC and Al5@NDC, respectively). Figure 1 shows the selected SEM images for Al4@NDC and Al5@NDC samples (for more SEM images, see ESI Figure S3). The synthesised Al-MOFs are perfectly homogeneous and are built of nano cuboids with an average size of their edge of 151 nm for Al5@NDC and 75 nm for Al4@NDC. Thus, in the presence of 5-aminotetrazole, crystallisation during the growth of MOF led to nano cuboids two times bigger than we used 4-aminotriazole. Furthermore, the size distribution of the nano cuboids is wider when the last is used as a crystallisation mediator. The obtained differences in morphology of the synthesised nanomaterials are not a consequence of embedding 5-amino tetrazole or 4-aminotriazole in the crystallography structure of MOFs, as composition analyses proved.

**Figure 2:**
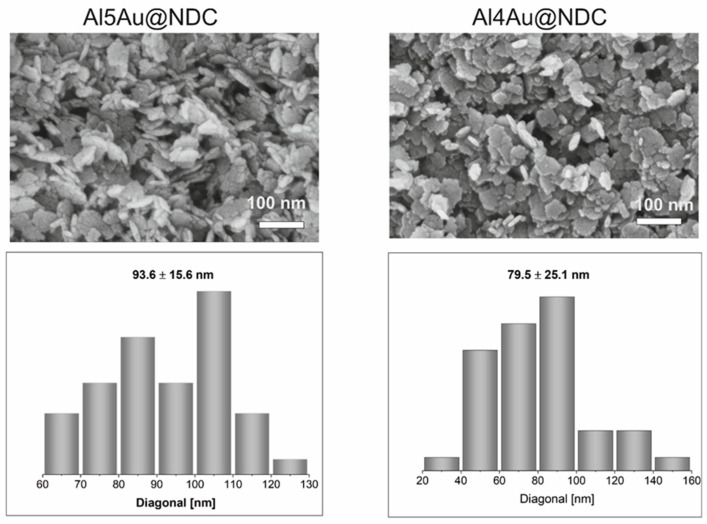
SEM images and histograms determined on the basis of these images’ composites: Al-MOF with AuNPs obtained using 5-amino tetrazole (Al5Au@NDC) and 4-amino triazole (Al4Au@NDC) as crystallisation mediators.

The composite nanomaterials were fabricated via embedding gold nanoparticles (AuNPs) in the structure of Al-based MOFs during the crystallisation. Firstly, AuNPs were prepared using a slightly modified Brust−Schiffrin procedure, producing ultra-small monodispersive nanoparticles (see ESI Figure S4). The growth of MOFs occurred in the presence of dissolved AuNPs and as earlier crystallisation mediators, 4-aminotriazole, or 5-amino tetrazole. The morphologies of the obtained nanocomposites, Al4Au@NDC and Al5Au@NDC, respectively, differ significantly from the MOFs fabricated without AuNPs. Figure 2 displays the selected SEM images for Al4Au@NDC and Al5Au@NDC composites. As shown in Figure 2, in the presence of AuNPs, the growth of MOFs leads to nanostructures with flake shape. These nanoflakes are more homogeneous when 5-amino tetrazole is used as a crystallisation mediator; they are bigger than those obtained using 4-aminotriazole.

The results of TEM analyses are consistent with those obtained from SEM and confirmed the nano cuboid structure of Al4@NDC and Al5@NDC and the nanoflakes morphology of their composites with AuNPs (see Figure S4 in the ESI). Additionally, it is visible in the composite material’s image that nanoparticles are embedded inside the nanocrystal structure.

The fabricated Al-MOFs and their composites with AuNPs have been analysed to determine their composition using a series of techniques: SEM with electro-dispersive spectroscopy (SEM-EDS), elemental analysis (E.A.), an inductively coupled plasma mass spectrometry (ICP MS), ^1^H NMR spectroscopy. Table 1 displays the content of elements determined from these techniques and the empirical formulas calculated.

ICP MS and SEM-EDS were used to determine aluminium and gold content, and notably, the results obtained are consistent, indicating that the materials are homogeneous. From E.A., the contents of carbon, hydrogen, nitrogen, and sulphur were determined. Although SEM-EDS also allows for determining carbon, nitrogen, and sulphur concentrations, results from E.A. are more accurate and were used to calculate empirical formulas.

The determined nitrogen content in the fabricated materials is low, 0.5–2.2% (*w*/*w*), indicating that the crystallisation mediator molecules are probably not built into the structures. Such ambiguities were unquestionably resolved by recorded ^1^H NMR spectra.

### 2.1. ^1^H NMR Spectroscopy

^1^H NMR spectra were recorded after the decomposition of MOFs in D_2_SO_4_ and dissolving in d6-DMSO. The spectra for both Al5@NDC and Al4@NDC are identical. There is a lack of proton signals from 4-aminotriazole and 5-amino tetrazole. There are visible signals from the linker, namely 1,4-naphthalene dicarboxylic acid. The signals in the range of 3.5–3.20 can be explained by DMF and water molecules coordinated in the MOF structure.

^1^H NMR (300 MHz, d6-DMSO) δ = 12.78 (s, COOH, 2H), 8.72–8.69 (m, CH, naphtalene ring 2H), 8.10 (s, CH, naphtalene ring, 2H), 7.67–7.64 (m, CH, naphtalene ring 2H), 3.54 (s, H_2_O), 3.35 (s, NH w (CH_3_)2NH), 3.20 (s, CH_3_, 6H).

While Al5@NDC and Al4@NDC do not differ in elemental composition there are minor differences in the measurement error range. There are slightly higher differences between Al5Au@NDC and Al4Au@NDC, especially in sulphur content (deriving from gold nanoparticles coating) and also for nitrogen and oxygen. However, the difference in catalytic activity of these materials (see further in the manuscript) cannot be explained based on their different compositions. In Table 1, empirical formulas are given and calculated based on their elemental composition. For all the materials, there is 1 mol of ligand per 1 mol of aluminium ions (NDC). Férey and co-workers [38] proved that hydroxyl ions are coordinated with metal ions in aluminium terephthalate MOF material (MIL-53). Our results of composition analyses indicate that in the aluminium-based MOFs linked with NDC, hydroxyl ions are also coordinated with aluminium ions in the same molar ratio (1:1 OH^−^:NDC^2−^). This conclusion is also consistent with the results from the structural investigation performed by Comotti et al. [35] for Al-based MOF with NDC obtained via the hydrothermal method.

From the nitrogen content, the molar content of DMF was calculated per 1 mol of aluminium ions. In the composite materials, the content of gold is 0.01 mol per 1 mol Al^3+^ ions.

### 2.2. Catalytic Assays

**Scheme 2:**
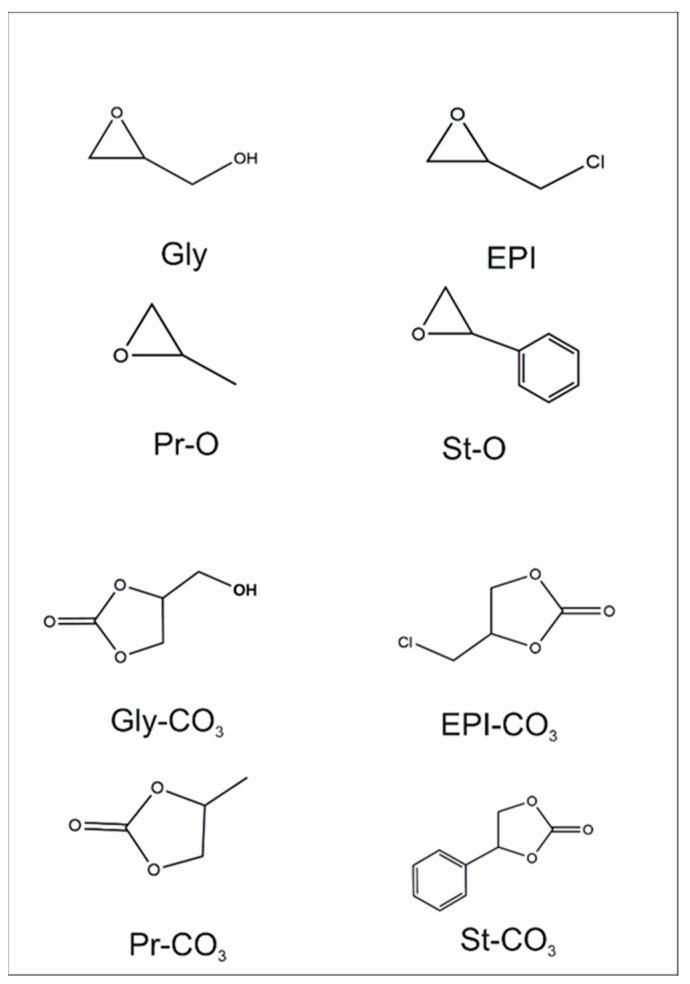
The formulas and abbreviations for the epoxides and products of their reaction with CO_2_ lead to the corresponding cyclic carbonates.

Scheme 2 shows formulas of epoxides tested in the cycloaddition of CO_2_ using the fabricated nanomaterials as catalysts and for the corresponding planned products.

Table 2 presents the results of catalytic assays performed for the model epoxides with all fabricated nanomaterials in the role of catalysts.

Employing the fabricated nanomaterials as catalysts in the cycloaddition of CO_2_ enables cyclic carbonates to be obtained for all tested epoxides under mild conditions (90 °C, 1 MPa CO_2_). The highest conversions and yields were obtained for glycerol carbonate (Gly-CO_3_), slightly smaller for reactions with epichlorohydrin and significantly lower for styrene and propylene oxide. For Al5@NDC, 100% conversion of glycidol was obtained within 4 h of conducting the reaction; extending the time to 22 h increases the reaction yield from 83% to 93%. This indicates that whole glycidol partially reacted to the transition product after 4 h, and can react further to the desired product. The embedded AuNPs in the structure of Al5@NDC allowed us to obtain a higher yield of Gly-CO_3_ for a significantly shorter reaction time. After 2 h of reaction, Gly with Al5Au@NDC conversion of the substrate is 91%, wherein the yield of Gly-CO_3_ is 89%, which indicates that the nanogold in the catalyst already at very low concentration (0.8%) allows for increasing selectivity. Kinetic measurements revealed that the reaction rate constant of cycloaddition CO_2_ to glycidol at 90 °C in the presence of Al5Au@NDC is 30% higher than with Al5@NDC (see Figure S2 in ESI).

In the reactions of cycloaddition CO_2_ to epichlorohydrin with the designed catalysts, 100% selectivity was obtained; however, the influence of gold in the catalysts on the course of reactions was not observed.

The reactions with styrene and propylene oxide occur with lower yields than with Gly and EPI. The lack of electron-withdrawing substituents in their molecules can explain the lower activity of these epoxides. For St-O, the highest conversion and comparable yield of St-CO_3_ were obtained using Al4@NDC (after 22 h reaction conducting conversion 28%, yield 27%). We do not observe the influence of gold in the catalysts on the yields of obtained St-CO_3_. In contrast, a significant impact was observed on the reaction with propylene oxide (Pr-O). In the presence of Al5@NDC, the yield of adding CO_2_ to Pr-O after 48 h is 18%; for Al5Au@NDC, the result is almost two times higher (34%).

**Figure 3:**
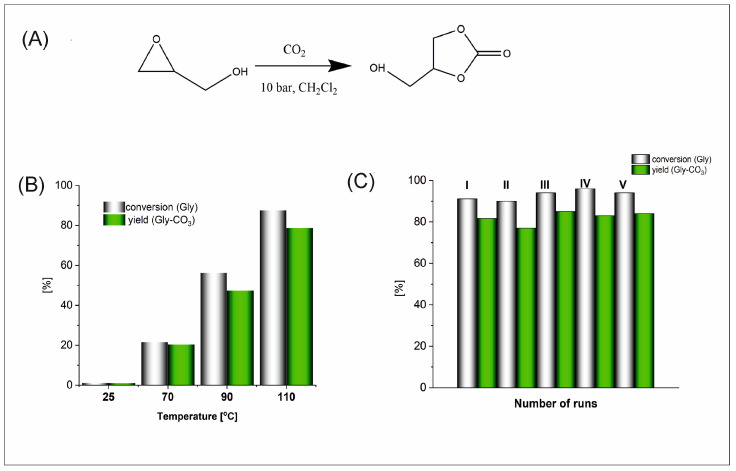
Scheme of CO_2_ cycloaddition to glycidol, (**A**); the influence of temperature on this reaction (1 h, Al5@NDC), (**B**); results for this reaction in five catalytic cycles with reused Al5@NDC (3 h, 90 °C, 20 mg of Al5@NDC used for the first cycle), (**C**).

The reaction conditions were optimised for the reaction of CO_2_ cycloaddition to glycidol, leading to primarily industrial product glycerol carbonate (Gly-CO_3_), and catalytic cycles were performed to evaluate the possibility of recycling the developed catalysts. Figure 3 shows the results of these assays for Al5@NDC, of which performance among the fabricated was most versatile.

Based on results obtained for glycidol, 90 °C was chosen as the optimal temperature in the cycloaddition reactions (Figure 3). At 70 °C, after 1 h of reaction, the conversion of Gly is three times smaller than at 90 °C; meanwhile, we compare 60% of conversion at 90 °C and 80% at 110 °C. The selection of a lower temperature is more economically justified, especially since this conversion after 1 h is satisfied.

Notably, the fabricated nanocatalysts can be easily separated from the post-reaction mixture (via centrifugation) and reused. This was proved by catalytic cycles performed for Al5@NDC for the cycloaddition reaction leading to Gly-CO_3_ (Figure 3). Over the course of five catalytic runs, we did not observe the deterioration of the conversion of Gly and the yield of obtained Gly-CO_3_. The minor differences are in the range of determination error.

The kinetic studies performed for the cycloaddition of CO_2_ to glycidol (relationships glycidol conversion and ln[Gly]_t_ vs. time), using the fabricated catalysts, showed that the first-order kinetic described this process very well (see Figure S2 in ESI). The determined reaction constant rates at 90 °C for the reaction using first-order kinetic simulations are given in Figure S2 (ESI).

In all catalytic assays, tetrabutylammonium bromide (TBAB) was used as a co-catalyst. We analysed the influence of the fabricated catalyst (Al5@NDC) and this co-catalyst (TBAB) separately and together on the cycloaddition of CO_2_ to glycidol, leading to glycerol carbonate. As it turned out, the reaction does not run without TBAB and Al5@NDC (conversion of the substrate was 0%); only 0.5% of conversion was obtained in the presence only Al5@NDC, 30% is achieved when TBAB is only used and almost two times higher when Al5@NDC and TBAB are used together (see Figure S5 in ESI).

### 2.3. Porosity Analyses—Isotherms of N_2_ and CO_2_ Adsorption

**Figure 4:**
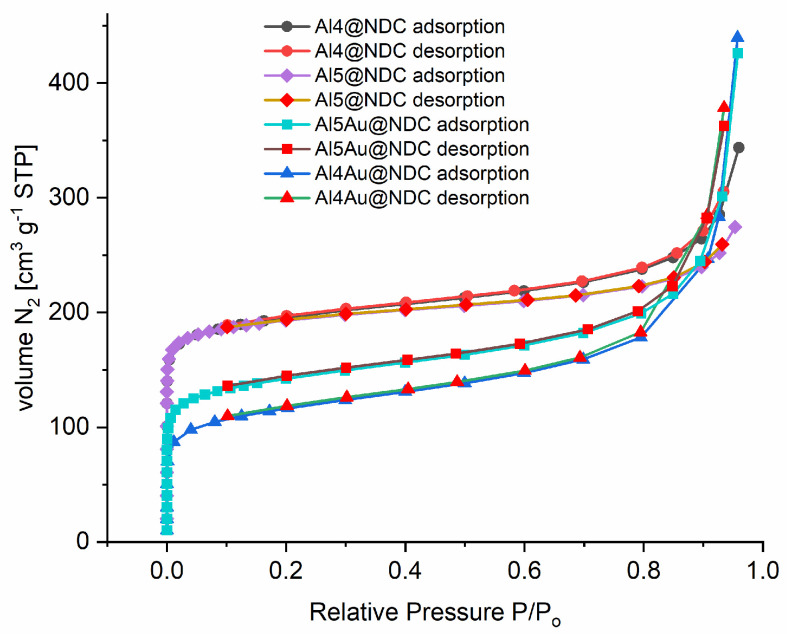
Porosity analyses of the fabricated nanocatalysts—N_2_ adsorption–desorption isotherms at 77 K.

Figure 4 shows isotherms of N_2_ adsorption recorded at 77 K for fourth fabricated nanocatalysts in adsorption and desorption cycles. Firstly, according to Brunauer classification [39], the perfectly reversible type II isotherms were recorded and indicated the microporous structure of the nanomaterials. The shape of curves is similar for all materials; nevertheless, significant differences in values of capacity for the adsorption of N_2_ given in volume per mass unit correlate with their ability to catalyse cycloaddition of CO_2_ to epoxides. All the studied materials are microporous with very high Brunauer–Emmett–Teller (BET) surfaces and an average pore diameter of 1 nm (Table 3). The most developed BET surface is for Al4@BDC and equals 741 m^2^ g^−1^; the lowest for Al4Au@NDC is 421 m^2^ g^−1^. Characteristic adsorption energy has been calculated for Al5@NDC (29 kJ mol^−1^). The isotherms of Al4@NDC and Al5@NDC are slightly different, consistent with results from SEM in their morphologies (see Figure 1). Al5@NDC is built of bigger nanocrystals, and its pores are smaller than in the case of Al4@NDC. For composite materials, namely Al5Au@NDC and Al4Au@NDC, porosity is lower than MOFs without gold nanoparticles, S_BET_ 526 m^2^ g^−1^ and 421 m^2^ g^−1^, respectively. It indicates that the nanoparticles occupy part of the pores inside the MOF structure, but these materials can still be classified as microporous (pore size up to 2 nm). Notably, the adsorption of N_2_ at 77 K is significantly higher for all fabricated materials than reported by Comotti et al. [35] for Al-based MOFs linked with NDC obtained via the solvothermal method. The difference in adsorption capability can be explained by bigger pores inside materials obtained from the DMF synthesis compared to the solvothermal process. As a result of the solvothermal procedure, pores with a width below 1 nm are formed (cross-sections 3 × 3 and 7.7 × 7.7 Å^2^). Meanwhile, in the MOFs fabricated according to our procedure are significantly bigger (see Figure S7 in ESI).

Table 3 shows characteristic parameters calculated on the base of N_2_ adsorption–desorption isotherms using the BET model and density functional theory (DFT).

The very high porosity of the fabricated nanocatalysts and small pore sizes in their structures enable more effective permeation of gases, which is crucial for achieving high yields in the cycloaddition of CO_2_.

**Figure 5:**
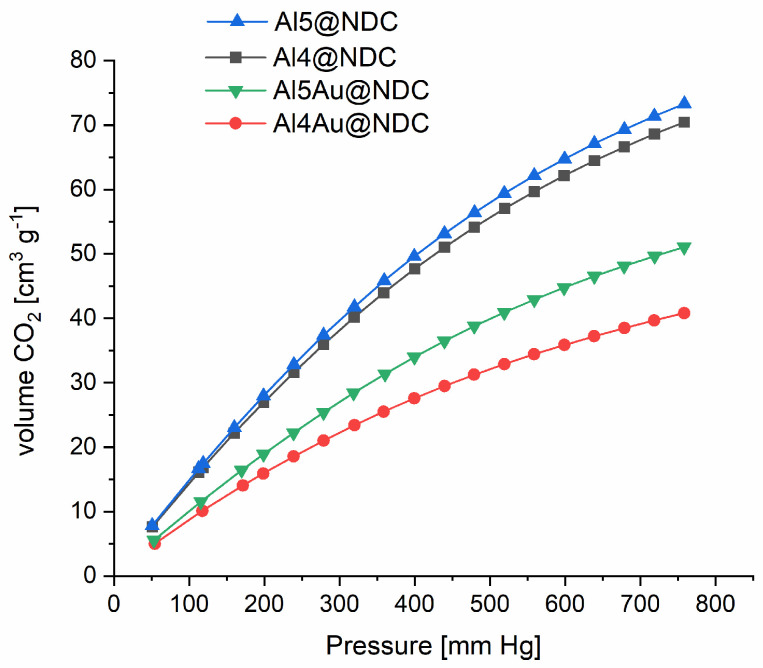
CO_2_ adsorption isotherms recorded for the fabricated nanocatalysts at 273 K.

Figure 5 displays the CO_2_ gas adsorption isotherms measured at 273 K for the synthesised nanomaterials. The results correlate perfectly with their catalytic activity in the cycloaddition of CO_2_ to epoxides.

Adsorption of CO_2_ increases with increasing pressure with steadily ascending slope patterns in the range of 50–800 mmHg for all synthesised nanomaterials. It indicates very strong adsorption of this gas on fabricated MOFs. For Al5@NDC, the CO_2_ adsorption capacity is highest and measured to be 3.125 mmol g^−1^ at 273 K and under normal pressure and is more than two times higher than recently reported for Co-MOFs with high catalytic performance in the cycloaddition of CO_2_ to epoxides [27]. Furthermore, the fabricated composite materials adsorb CO_2_ with lower capacity, and for Al4Au@NDC, the lowest adsorption among all the materials is 1.562 mmol g^−1^. Nevertheless, compared with others reported in the literature, composite materials of this type have high capacities. Importantly, considering the obtained results, the increased catalytic activity of Al5Au@NDC in cycloaddition CO_2_ to propylene oxide compared with Al5@NDC cannot be explained by higher gas adsorption by the first mentioned catalyst. It proves that the participation of gold nanoparticles in the catalytic process is responsible for the observed differences between Al5@NDC and Al5Au@NDC. Meanwhile, the better catalytic performance of Al5Au@NDC compared to Al4Au@NDC may be explained by the higher CO_2_ adsorption capacity of the first.

### 2.4. Thermogravimmetric Analyses (TGA)

TGA allowed us to evaluate the fabricated nanocatalysts’ thermal stability and analyse their composition changes under heating.

**Figure 6:**
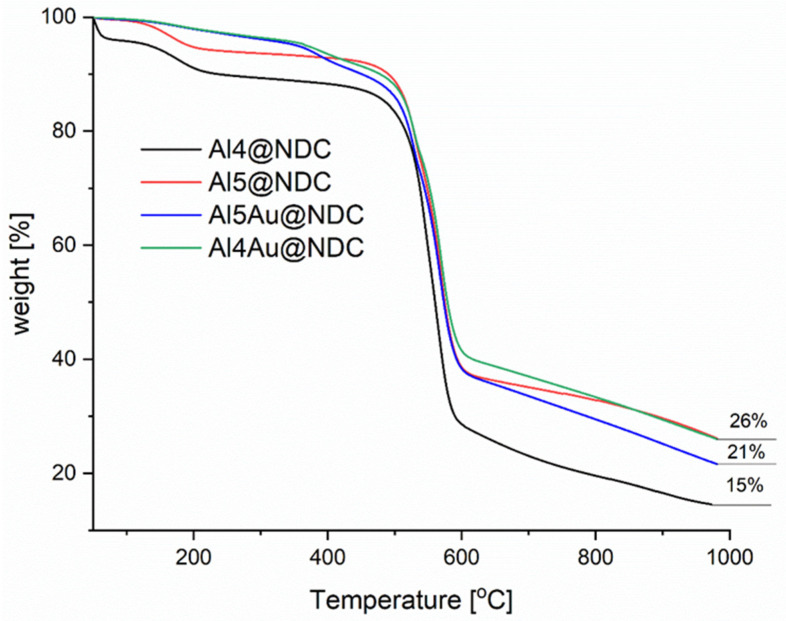
TGA curves recorded for the fabricated nanocatalysts.

Figure 6 shows TGA curves recorded during the heating up to 1000 °C under a nitrogen atmosphere with a heating ratio of 10 K min^−1^.

Two weight losses are observed in the TGA curve measured for Al5@NDC and Al4@NDC. First, small in the range of 150–200 °C corresponding with residue DMF molecules releasing from the structure of these MOFs (is ca. 7% of starting mass in both cases, which corresponds very well with 6% of DMF predicted for the materials from composition analyses, see in Table 1) and significantly higher in the range of 500–600 °C related to thermal decomposition of these materials. The thermal decomposition of Al4@NDC begins at a lower temperature than Al5@NDC and leads to a lower residue mass (15% of the starting mass). This residue mass is aluminium and carbon from the decomposition of organic matter under anaerobic conditions.

TGA curves registered for composite materials, namely Al5Au@NDC and Al4Au@NDC, are very similar. Thermal decomposition begins at a slightly lower temperature than Al5@NDC and Al4@NDC, which do not contain AuNPs. The decay of the ligand coating AuNPs (1-octadecanethiol) is probably visible as the step before the MOFs network decomposes.

TGA showed high thermal stability for Al-MOFs and their nanocomposites with gold nanoparticles; they are stable up to 450 °C, making them good candidates for catalysts that also work at high temperatures.

### 2.5. Powder X-ray Diffraction (PXRD) Analyses and Crystal Structure Simulations

**Figure 7:**
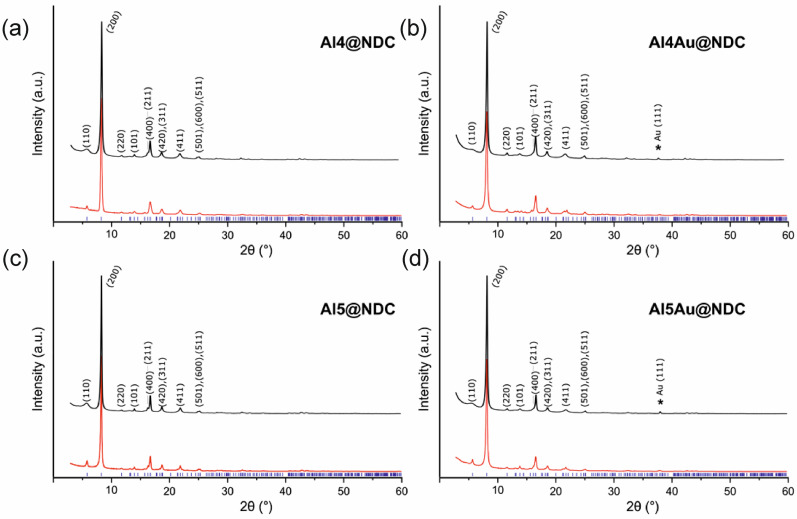
Powder X-ray diffraction (PXRD) patterns of MOF nanomaterials (**a**,**c**) and their composites with AuNPs (**b**,**d**) after drying (the reflex originating from Au is marked with an asterisk). Experimental patterns and the corresponding fits to the tetragonal P4/nmm crystallographic system are shown in black and red, respectively.

Powder X-ray diffraction (PXRD) patterns of the MOFs shown in Figure 7 (black curves) indicate a high degree of crystallinity despite the fine microstructure revealed by the SEM and TEM imagining (Figure 1 and Figure S4). Al4@NDC (Figure 7a,b) and Al5@NDC (Figure 7c,d) lattices, as indicated by a high-level similarity of their diffraction patterns, regardless of the presence of AuNPs, conform to the same tetragonal P4/nmm crystallographic system with unit-cell parameters listed in the Table S1 (ESI). We note that high-temperature or extended period room-temperature drying of the MOFs leads to the emergence of (110) reflexes absent in the as-synthesized materials (Figure S8). It also helps in matching the powder patterns to previously reported MOF whose crystallographic structure has been resolved from synchrotron data [35]. Miller indices of the most prominent MOF reflexes are shown above the experimental patterns, while the red curves at the bottom of each panel in Figure 7 show the best fit to the data, along with predicted reflexes positions (blue ticks). The presence of gold nanoparticles in MOF Al4Au@NDC (Figure 7b) and MOF Al5Au@NDC (Figure 7d) is manifested by the presence of weak reflex at 38.5° (2Θ) originating from (111) face-centred cubic lattice planes of Au marked with an asterisk. We note that these reflexes likely originate from the largest nanoparticles present in the samples.

### 2.6. Small-Angle X-ray Scattering (SAXS) Analyses of AuNPs

Small-angle X-ray scattering patterns of thiol-coated gold nanoparticles dispersed at 1 wt. % in toluene were acquired. The analysis of scattering profiles indicated bimodal size distribution model consisting of small and large nanoparticles (see Figure S6 in ESI) with an average diameter of 3.7 nm and 22.1 nm, respectively. It indicates the presence of the main fraction of very small nanoparticles visible in the TEM images (see Figure S4 in ESI) and the second, minority fraction of larger nanoparticles or particle aggregates. However, considering the pore size in the fabricated MOF materials (see discussion 2.3), only the fractions of smaller N.P.s can be incorporated into the voids of the composite and larger nanoparticle particles, detectable in PXRD, are likely present on the outside or in the defects of MOF crystalline lattice.

## 3. Materials and Methods

### 3.1. Materials

Al(NO_3_)_3_ • 9H_2_O (Merck, Warsaw, Poland), ACS reagent, ≥98% (Merck, Poland), 1,4-naphtalene dicarboxylic acid (1,4-NDC), 98% (AmBeed Inc., Arlington, IL, USA), HAuCl_4_ • 3H_2_O), ACS reagent, ≥49.0% Au basis (Merck, Poland), 1-octadecanethiol ≥98% (Merck, Poland), tetraoctylammonium bromide [CH_3_ (CH_2_)_7_]_4_NBr TOAB, 98% (Merck, Poland), tetrabuthylammonium bromide ([CH_3_ (CH_2_)_3_]_4_NBr) TBAB, 98% (Merck, Poland), NaBH_4_, powder ≥ 98% (Sigma Aldrich, Poznań, Poland), glycidol, (Gly) 96% (Thermo Scientific, Bionovo, Legnica, Poland), Epichlorohydrine (EPI), 99% (Thermo Scientific, Bionovo, Poland), Styren oxide (St-O) ≥ 97% (Thermo Scientific, Bionovo, Poland), (+/−) Propylene oxide (Pr-O), ≥99% (Thermo Scientific, Bionovo, Poland), dimethylformamide DMF (>95%) (Chemat, Gdansk, Poland), dichlormethane (DCM) > 95%, (Chemat, Poland), ethyl acetate > 95%, (Chemat, Poland), toluene (>95%) (Chemat, Poland), ethanol anhydrous (≥99%) (Chemat, Poland), 4H-1,2,4-triazol-4-amine (4-Atrz), 95% (AmBeed, Inc., Arlington, USA), 5-amino-1H-tetrazole (5-Atz), 95% (Angene Chemical, Hyderabad, India), anizole anhydrous, 99.7%, (Merck, Poland), propylene carbonate, anhydrous 99.7%, (Merck, Poland), 4-(Hydroxymethyl)-1,3-dioxolan-2-one, ≥98%, (Merck, Poland), methanol anhydrous, 99% (Merck, Poland).

### 3.2. Synthesis of Al4@NDC/Al5@NDC

An amount of 92.4 mg of Al(NO_3_)_2_ • 9H_2_O (0.25 mmol), 16.8 mg (0.2 mmol) of 4-Atrz (in the case of Al4@NDC) or 17 mg (0.2 mmol) of 5-Atz (in the case of Al5@NDC), and 86.5 mg of 1,4-NDC (0.4 mmol) were placed in a vial and dissolved in 10 mL of DMF. The solution was stirred for 10 min using a magnetic stirrer; after this, the solution was completely clear. The closed vial was heated in an oven for 72 h at 110 °C.

Afterwards, the precipitated crystals were filtered, thoroughly rinsed with methanol and dried under an argon atmosphere. The dried crystals were transferred to the vial, anhydrous methanol was added (10 mL), and the tightly closed vial was shaken using an open-air lab shaker for 24 h (100 rpm). After this time, the mixture was centrifuged (1000 rpm, 10 min), the supernatant was discarded and solid dried in an oven for 24 h at 165 °C. White, fine crystalline products (60 mg of Al5@NDC and 55 mg of Al4@NDC) were obtained.

### 3.3. Synthesis of AuNPs

AuNPs stabilised by 1-octadecanethiol were synthesised according to the Brust−Schiffrin [40] procedure, wherein it was modified to obtain the product in a scale of grammes.

The solution, which was obtained by dissolving 100 mg of HAuCl_4_ • 3H_2_O (5 mmol) in 120 mL of ultra-pure water, was extracted with a solution of TOAB in toluene (1.53 g of TOAB in 180 mL) three times. The toluene layer became deep orange, and the aqueous layer was discarded. The toluene solution was transferred to the flask placed on a magnetic stirrer, 673 mg of 1-octadecanethiol dissolved in 25 mL of toluene was added, and when the obtained solution was mixed very intensively, the solution of NaBH_4_ in methanol (400 mg NaBH_4_, 100 mL MeOH) was added dropwise. After the first several drops of NaBH4 solution_,_ the mixture became black. The mixture was stirred for 20 h at room temperature.

Afterwards, the post-reaction mixture was extracted with ultra-pure water (2 × 200 mL), water layers were discarded, the organic layer was concentrated using a rotary vacuum evaporator to ca. 10 mL and 50 mL of cooled down ethanol (96%) was added to precipitate the obtained nanoparticles. The suspension was centrifuged (15,000 rpm, 5 min), the supernatant was discarded, and the solid product was dried in a vacuum oven for 24 h (40 °C, 10 mbar). The final product (1.0342 g) was a black powder soluble in most organic solvents.

### 3.4. Synthesis Al5Au@NDC/Al4Au@NDC

Syntheses of composite materials with MOFs were performed following a strategy called “ship in a bottle” [41]. The MOF network is built around the nanoparticles dissolved in the solution.

An amount of 26 mg AuNPs (0.05 mmol Au^3+^), 92.4 mg (0.25 mmol) Al(NO_3_)_3_ • 9H_2_O, 16,8 mg (0.2 mmol) 4-Atrz (in the case of Al4Au@NDC) or 17 mg (0.2 mmol) 5-Atz (in the case of Al5Au@NDC), and 86.5 mg 1,4-naphthalene dicarboxylic acid (1,4-NDC) (0.4 mmol) were dissolved in 10 mL of DMF. The solution was stirred for 10 min using a magnetic stirrer; after this, the solution was completely clear. The closed vial was heated in an oven for 72 h at 110 °C.

Afterwards, the precipitated crystals were filtrated, thoroughly rinsed with methanol and dried under an argon atmosphere. The dried crystals were transferred to the vial, anhydrous methanol was added (10 mL), and the tightly closed vial was shaken using an open-air lab shaker for 24 h (100 rpm). After this, the mixture was centrifuged (1000 rpm, 10 min), the supernatant was discarded, and the solid dried in an oven for 24 h at 165 °C. Slightly pink, fine crystalline products were obtained (80 mg of Al5Au@NDC and 85 mg of Al4Au@NDC).

### 3.5. Catalytic Assays

The reactions of cycloaddition CO_2_ in the presence of the fabricated catalysts were performed in the pressure reactor constructed in our lab using commercially available elements made of stainless steel. The reactor has a manometer and valve system to introduce CO_2_ at proper pressure. Reagents were placed in a Teflon vessel inside the reactor, and CO_2_ was introduced from a cylinder to start pressure 1 MPa. The reactor was immersed in a thermostated (±2 °C) oil bath on a magnetic stirrer (see Figure S1 in ESI).

The reactions were performed in two systems:

- I. In solution with DCM
- II. Without a solvent on a four times bigger scale than I.

I. In a teflon vessel, 10 mg of catalyst was placed, and the solution which was obtained by dissolving 4 mmol epoxide, 29 mg TBAB (0.09 mmol) and ca. 80 mg of anisole (internal reference for G.C. analysis) in 6 mL DCM. The vessel was closed in the reactor. Next, CO_2_ was introduced to the reactor to start pressure 1 MPa. The reactor was immersed in a thermostated oil bath set at a temperature on a magnetic stirrer.
II. In a teflon vessel, 30 mg of catalyst was placed, and the solution which was obtained by dissolving 15 mmol epoxide in the case of Gly (ca. 1 mL), 20 mmol in the case of EPI, 200 mg TBAB (0.732 mmol) and ca. 280 mg of anisole (internal reference for G.C. analysis). The vessel was closed in the reactor. Next, CO_2_ was introduced to the reactor to start pressure 1 MPa. The reactor was immersed in a thermostated oil bath on a magnetic stirrer.

After a selected time, the reactor was cooled down using ice water, degassed, and the post-reaction mixture was mixed with 7 mL of ethyl acetate (this made it easier to separate the catalyst). Afterwards, the obtained suspension was filtrated using a syringe filter (200 µm) or, in the case of catalyst recycling experiments, the catalyst was centrifuged from the post-reaction mixture (10 min, 10,000 rpm), allowing the catalyst to be reused. The recovered catalyst was washed with methanol, dried at 50 °C for 24 h, and reused. The mixture obtained after catalyst separation was directly analysed using gas chromatography (G.C.). Analyses of the substrate conversion and product yields were performed using an internal standard procedure with anisole as the internal standard added to the reaction mixture; for the selected post-reaction mixture, product identity was confirmed by ^1^H NMR spectroscopy.

### 3.6. Techniques

#### 3.6.1. Gas Chromatography (G.C.)

G.C. analyses to determine yields and conversions of reactions with CO_2_ were performed using a Shimadzu GC2010 Plus gas chromatograph (Shimadzu Corp., Kyoto, Japan) equipped with a split-mode capillary injection system and flame ionisation detector (FID) using capillary column: HP-5 (30 m × 0.320 mm, 0.25 μm, (Shimpol, Warszawa, Poland).

Chromatography conditions: carrier gas—nitrogen (~80 kPa); split ratio: 50; injector temperature 250 °C; detector temperature 280 °C.

G.C. analysis method for the reaction of styrene oxide or propylene oxide with CO_2_:

Pressure: 80.9 kPa; Column flow: 2.43 mL/min; oven temperature: initial temp. 80 °C hold for 2 min; ramp rate 40 °C/min to 280 °C; final temp. 280 °C hold for 5 min (12 min total).

G.C. analysis method for the reaction of glycidol or epichlorohydrin with CO_2_:

Pressure: 77.3 kPa; column flow: 2.54 mL/min; oven temperature: initial temp. 60 °C hold for 2 min; ramp rate 40 °C/min to 280 °C; final temp. 280 °C hold for 5 min (12.5 min total).

#### 3.6.2. Scanning Electron Microscopy with Electron Dispersive Spectroscopy (SEM-EDS)

The JEOL-JSM-5600 microscope with OXFORD Link–ISIS–300 spectrometer (Zeiss, Jena, Germany) was used to image the morphology and elemental composition of the fabricated nanocatalysts. For imaging, the samples were coated with a thin layer of Au–Pd alloy to improve conductivity.

#### 3.6.3. Transmission Electron Microscopy (TEM)

Transmission electron microscopy (TEM) observations have been carried out using a JEM 1400 JEOL Co. microscope (Zeiss, Germany) at 120 kV acceleration voltage. The samples were obtained by casting the DMF solutions of materials onto a carbon-coated copper microgrid (200 mesh) and air-dried overnight.

#### 3.6.4. Porosity Analyses

The adsorption–desorption of nitrogen (N_2_) and carbon dioxide (CO_2_) isotherms were measured, and the specific surface area and pore volume using Micrometrics ASAP 2020 (Norcross, GA, USA). Before measuring, each sample was heated at 200 °C for 6 h; next, pressure was decreased to achieve <1 µm Hg. The measurements of N_2_ adsorption were conducted at 77 K, while the adsorption of CO_2_ was at 273 K. The Brunauer–Emmett–Teller (BET) equation was used to calculate the surface area. The pore volumes and their surfaces were calculated using density functional theory (DFT) methods.

#### 3.6.5. Thermogravimetric Analyses (TGA)

T.G. analyses were conducted under an N_2_ atmosphere with a heating rate of 10 °C min^−1^ using a T.A. Instruments Q50 V20.10 Build 36 (T.A. Instruments, New Castle, DE, USA) thermogravimetric analyser.

#### 3.6.6. NMR Spectroscopy

^1^H NMR spectra of the fabricated MOFs were recorded after decomposition in deuterated sulphuric acid (100%) and dissolving in DMSO-d_6_.

^1^H NMR spectra of post-reaction mixtures were recorded in CDCl_3_.

All spectra were recorded using Bruker Corporation 300 MHz spectrometer.

The samples of MOFs with mass ca. 3 mg were first dissolved in 20 µL D_2_SO_4_ (100%), 600 µL DMSO-d_6_ was added, and the suspension was sonicated for 10 min before recording a spectrum.

#### 3.6.7. Powder X-ray Diffraction (PXRD) and Small-Angle X-ray Scattering (SAXS)

Diffractograms of MOFs deposited on <911>-cut zero-background silicon wafers were collected using a powder X-ray diffractometer (D8 Discover, Bruker Inc., Billerica, MA, USA) equipped with a line-shaped collimated Cu Kα radiation (0.154 nm) source in 3–60° 2θ range in a locked-coupled mode. The position and relative intensities of the 20 most prominent reflexes were used to determine the most plausible space group and unit-cell parameters of the material using the Expo2014 PXRD data analysis software (https://www.ba.ic.cnr.it/softwareic/expo/) [42].

Small angel X-ray scattering patterns were collected over 1200 s period using a 2D gas-wire detector (Vantec 2000, Burgebrach, Germany) in 0.1–5° 2θ scattering angle range and, subsequently, azimuthally integrated using silver behenate as a calibrant to obtain the scattering intensity dependence on the scattering vector q = 4π/λ sinθ, where λ = 1.542 nm is the X-ray wavelength. All SAXS patterns were radially averaged and corrected for background scattering. The data were analysed with SasViev software (https://www.sasview.org/) [43].

#### 3.6.8. Elemental Analyses

The elemental UNIcube apparatus was used to analyse the MOF samples and composite materials (C, H, N, S).

#### 3.6.9. An Inductively Coupled Plasma Mass Spectrometry (ICP MS)

An inductively coupled plasma mass spectrometer, ICP-MS, NexION 300D (Perkin Elmer, Boston, MA, USA), equipped with a quartz cyclonic spray chamber and Meinhard nebuliser, was used for Al and Au concentration measurements.

## 4. Conclusions

Novel aluminium-based MOF materials were fabricated using 1,4-naphthalene dicarboxylic acid as a linker and 4-aminotriazole and 5-aminotetrazole as crystallisation mediators. The composite materials of Al-based MOFs with embedded gold nanoparticles (AuNPs) were also prepared using the “ship-in-a-bottle” strategy.

Al-based MOFs form cube/cuboid-shaped nanostructures, wherein the composite materials form nanoflakes. Both structures characterise narrow size dispersity. PXRD studies proved the fabricated materials’ high degree of crystallinity with the same tetragonal P 4/nmm crystallographic system with slightly differing unit-cell parameters.

The synthesised materials are highly porous (S_BET_ in the range 421 m^2^ g^−1^ for Al4Au@NDC do 735 m^2^ g^−1^ for Al5@NDC), homogeneous, and thermal stable up to 450 °C. They exhibit high CO_2_ adsorption capacity of up to 3.125 mmol g^−1^ at 273 K and under normal pressure.

The fabricated materials, in combination with TBAB as a co-catalyst, give good and excellent catalytic activity for the carbon dioxide cycloaddition with a series of model epoxides: epichlorohydrin, glycidol, styrene and propylene oxide. Applying the synthesised catalysts enables the reactions to be performed under mild conditions (90 °C, 1 MPa CO_2_) for a short time with high conversion and yield (91% conversion of glycidol towards glycerol carbonate with 89% yield within 2 h). The developed nanocatalysts can be easily separated from the reaction mixture and reused several times without deteriorating performance.

The presence of AuNPs in the synthesised nanocatalysts (0.8% *w*/*w*) enables the cycloaddition of CO_2_, leading to cyclic esters with higher yields for a shorter time. This phenomenon is evident for less-active epoxides like propylene oxide, for which a two times higher yield of propylene carbonate was obtained using nanocomposites compared to Al-MOF without nanoparticles.

The designed Al-MOFs and their composites with AuNPs give good and excellent results in the cycloaddition of carbon dioxide to tested model epoxides. Considering the prices and commercial availability of reagents needed for their preparation, ease of obtaining, the safety of these materials for the environment and thermal stability, they seem perfect candidates for catalysts for the sustainable fixation of CO_2_ on an industrial scale.

## Figures and Tables

**Table 1:** The fabricated nanomaterials’ elemental composition (given in % *w*/*w*) and calculated their empirical formulas.

Al	C	N	S	H	O	Au
Al5@NDC						
8.65 ^a^ 8.04 ^d^	50.1 ^b^ 54.5 ^d^	0.99 ^b^ 2.24 ^d^	-	3.96 ^b^	36.3 ^c^ 35.2 ^d^	-
Formula	Al (OH)(C_12_H_6_O_4_) • 0.22 (CH_3_)_2_NC(O)H • 2 H_2_O AlOH (NDC) • 0.22 (DMF) • 2H_2_O					
Al5Au@NDC						
8.57 ^a^ 8.33 ^d^	50.8 ^b^ 57.5 ^d^	0.61 ^b^ 1.64 ^d^	0.57 ^b^ 0.71 ^d^	3.97 ^b^	35.5 ^c^ 30.9 ^d^	0.82 ^a^ 1.17 ^d^
Formula	Al (OH)(C_12_H_6_O_4_) • 0.14 (CH_3_)_2_NC(O)H • 0.05 S • 0.01 Au • 2 H_2_O AlOH (NDC) • 0.14 (DMF) • 0.05 S • 0.01 Au • 2 H_2_O					
Al4@NDC						
8.76 ^a^ 4.38 ^d^	50.3 ^b^ 53.0 ^d^	1.12 ^b^ 2.21 ^d^	-	4.07 ^b^	35.7 ^c^ 40.4 ^d^	-
Formula	Al (OH)(C_12_H_6_O_4_) • 0.25 (CH_3_)_2_NC(O)H • 2 H_2_O AlOH (NDC) • 0.25 (DMF) • 2 H_2_O					
Al4Au@NDC						
8.59 ^a^ 7.08 ^d^	51.6 ^b^ 59.5 ^d^	0.57 ^b^ 1.73 ^d^	1.02 ^b^ 1.09 ^d^	4.05 ^b^	34.2 ^c^ 27.5 ^d^	0.85 ^a^ 0.59 ^d^
Formula	Al (OH)(C_12_H_6_O_4_) • 0.13 (CH_3_)_2_NC(O)H • 0.1 S • 0.01 Au • 2 H_2_O AlOH(NDC) • 0.13 (DMF) • 0.1 S • 0.01 Au • 2 H_2_O					

^a^ ICP MS; ^b^ EA; ^c^ calculated as 100%—(Al% ^a^ + C% ^b^ + N% ^b^ + H% ^b^); ^d^ SEM-EDS, NDC—1,4-naphtalene dicarboxylic acid (deprotonated), DMF—dimethyloformamide.

**Table 2:** Results of cycloaddition CO_2_ to epoxides using the fabricated nanocatalysts (formulas given in Scheme 2) ^a^.

Substrate	Product	Time [h]	Conversion [%]	Yield [%]	TON
Al5@NDC					
Gly	Gly-CO_3_	22 20 ^b^ 4 2	100 87 100 82	94 79 83 70	117 114 100 84
EPI	EPI-CO_3_	18 6 2	91 62 22	91 62 22	63 42 15
Pr-O	Pr-CO_3_	48	n.d.	18	14
St-O	St-CO_3_	22	11	7	5
Al5Au@NDC					
Gly	Gly-CO_3_	18 4 2	100 100 91	88 88 89	102 103 104
EPI	EPI-CO_3_	18	91	90	61
Pr-O	Pr-CO_3_	22 48	n.d. n.d.	17 34	7 10
St-O	St-CO_3_	22	19	16	11
Al4@NDC					
Gly	Gly-CO_3_	19 4 2	100 91 84	80 77 73	95 95 82
EPI	EPI-CO_3_	2 6 20 ^c^	22 62 93	22 62 93	15 42 166
Pr-O	Pr-CO_3_	48	n.d.	14	11
St-O	St-CO_3_	22	28	27	15
Al4Au@NDC					
Gly	Gly-CO_3_	18	100	89	103
EPI	EPI-CO_3_	18	94	94	65
Pr-O	Pr-CO_3_	22	n.d.	5	2
St-O	St-CO_3_	22	24	23	16
AuNPs					
St-O	St-CO_3_	24	19	9	n.c.

^a^ Reaction conditions: 90 °C, 1 MPa CO_2_, 6 mL DCM, 10 mg of catalyst, 29 mg of TBAB (co-catalyst), ^b^ without solvent in a scale 15 mmol of Gly, 32 mg of Al5@NDC and 200 mg of TBAB, ^c^ without solvent in a scale 19.8 mmol of EPI, 33 mg of Al5@NDC and 250 mg of TBAB, n.d.—not determined due to the high volatility of propylene oxide; thus, it was not possible to analyse this compound using G.C., TON—the turnover number defined as n_product_/n_catalyst_ (molar mass of catalyst was calculated on the base of the formula given in Table 1), n.c.—not calculated as the molecular weight of AuNPs has not been determined.

**Table 3:** Porosity parameters calculated from N_2_ adsorption–desorption isotherms at 77 K using the BET model and density functional theory: S_BET_—surface area from BET, S_DFT_—surface area from QSDFT, L_o_—pore size according to Stoecklicky, E_0_—Dubinin energy of adsorption.

	S_BET_ [m^2^ g^−1^]	S_DFT_ [m^2^ g^−1^]	L_o_ [nm]	E_o_ [kJ mol^−1^]
Al5@NDC	736.5	834.9	0.61	29.02
Al4@NDC	741.3	827.6	0.72	26.38
Al5Au@NDC	526.4	595.7	0.84	24.20
Al4Au@NDC	421.0	463.5	1.05	21.70

## Data Availability

Data are contained within the article and Supplementary Materials.

## Supplementary Materials

The following supporting information can be downloaded at: https://www.mdpi.com/article/10.3390/ijms25021020/s1. References [42,44] are cited in the Supplementary Materials.
