# Supplementary material for: Aluminium-Based Metal–Organic Framework Nano Cuboids and Nanoflakes with Embedded Gold Nanoparticles for Carbon Dioxide Fixation with Epoxides into Cyclic Esters
Source: Int J Mol Sci. 2024 Jan 13;25(2):1020. doi: 10.3390/ijms25021020 (PMC10816805; doi:10.3390/ijms25021020)
Supplement: Supplementary file 1 [file ijms-25-01020-s001.zip › ijms-2808057-supplementary.pdf]

Supplementary Materials

Aluminium-based MOF nanocubes and nanoflakes with embedded gold nanoparticles for carbon dioxide cycloaddition to epoxides

Gabriela Kopacka[1], Kinga Wasiluk[1], Paweł W. Majewski[1,2], Michał Kopyt[1,2], Piotr Kwiatkowski[1,2] and Elżbieta Megiel[1*]

[1]Faculty of Chemistry, University of Warsaw, Pasteur 1, PL 02-093 Warsaw, Poland

[2] Biological and Chemical Research Centre, University of Warsaw, Żwirki i Wigury 101, 02-089 Warsaw, Poland

Table of Contents:

Figure S1. The picture of the reaction set used in the catalytic assays.

Figure S2. Kinetic curves of the cycloaddition CO_2 to glycidol catalyzed by the fabricated catalysts. Relationship conversion vs. time for the given catalyst is drawn in the same colour as the first-order kinetic simulation curve. Reaction conditions: 90°C, 1 MPa CO_2, 10 mg catalyst, 29 mg TBAB, 6 ml DCM.

Figure S3. The selected SEM images of the fabricated catalysts.

Figure S4. The selected TEM images of the fabricated materials.

Figure S5. Influence of the fabricated catalyst (Al5@NDC) and cocatalyst (TBAB) separately and together on the cycloaddition of CO_2 to glycidol leading to glycerol carbonate (time of reaction 1 h, t=90°C, 1 MPa CO_2).

Additional information on PXRD analyses**.**

Figure S6. SAXS pattern recorded for the AuNPs solution in toluene.

Table S1 Structural details from PXRD simulations

Figure S7. The distribution of pore sizes in the fabricated MOF materials determined from N2 adsorption analyses.

Figure S8. Powder X-ray diffraction (PXRD) patterns of as-synthesized MOF nanomaterials: a) Al4@NDC, b) Al4@NDC, c) Al5@NDC, and d) Al5Au@NDC.

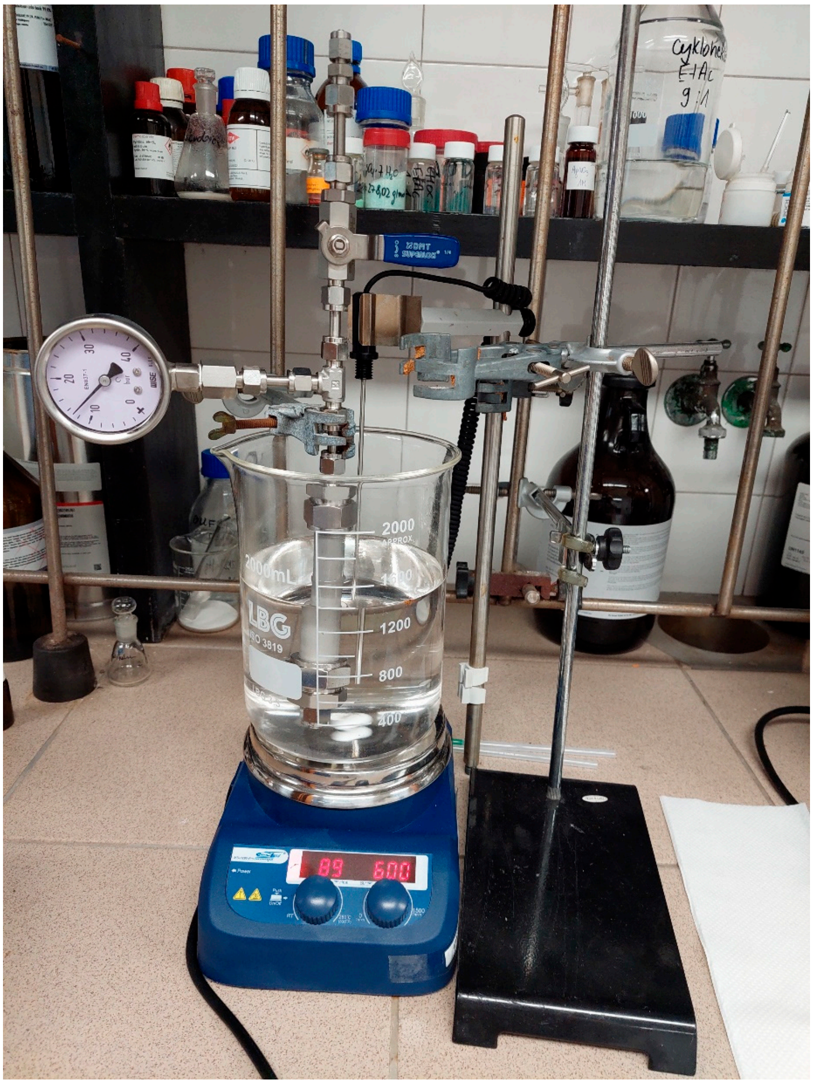

Figure S1. The picture of the reaction set used in the catalytic assays.

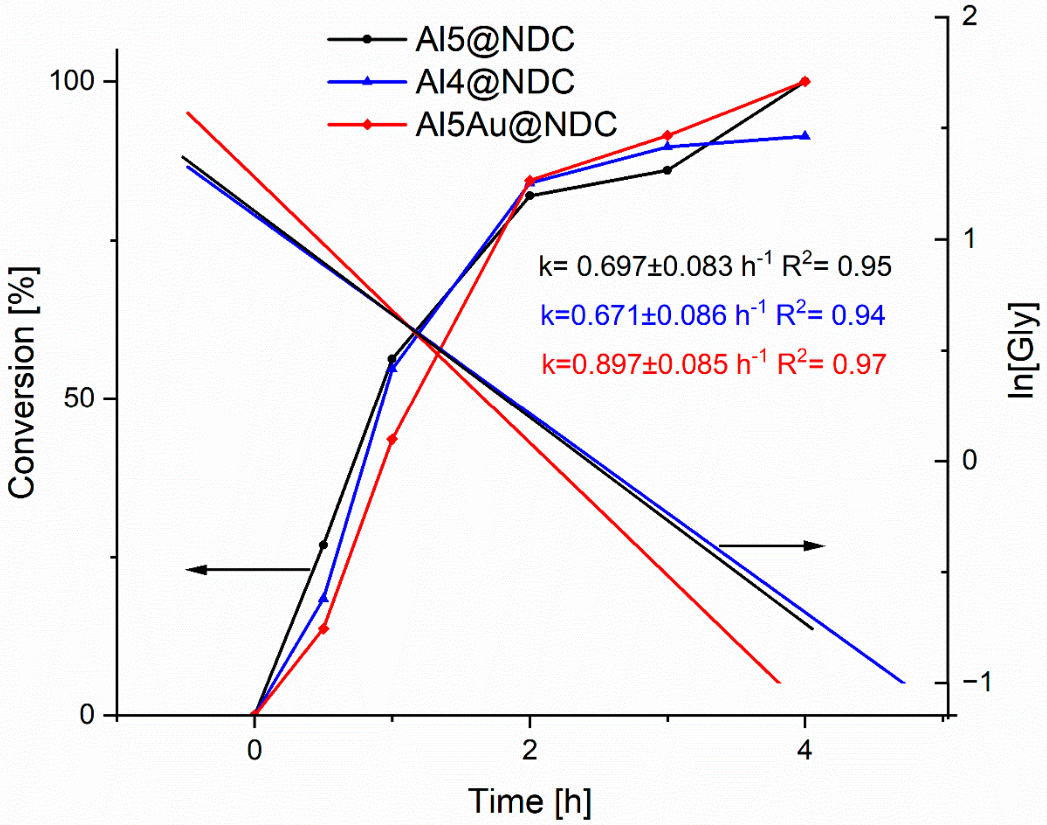

Figure S2. Kinetic curves of the cycloaddition CO_2 to glycidol catalyzed by the fabricated catalysts. Relationship conversion vs. time for the given catalyst is drawn in the same colour as the first-order kinetic simulation curve. Reaction conditions: 90°C, 1 MPa CO_2, 10 mg catalyst, 29 mg TBAB, 6 ml DCM.

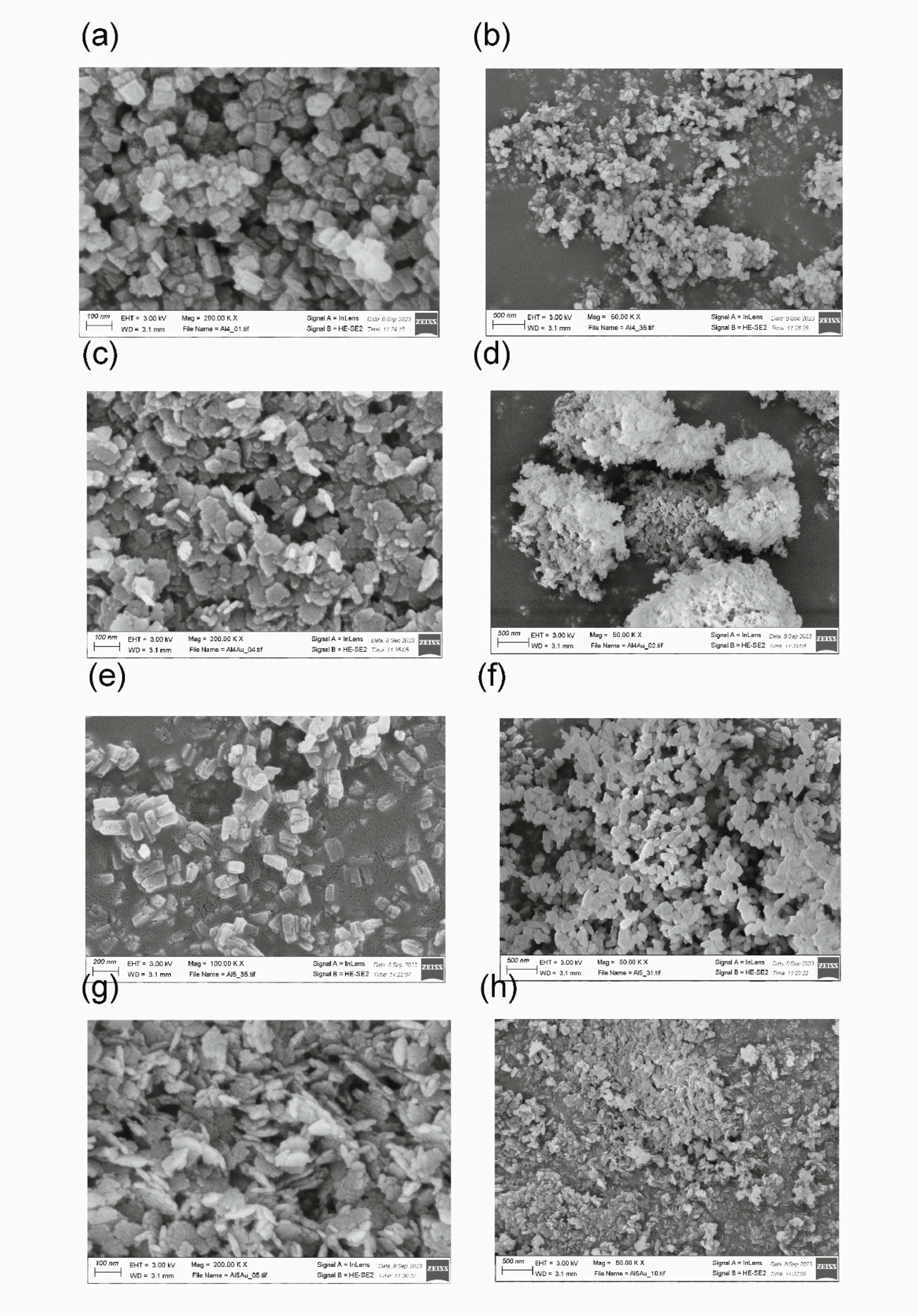

Figure S3. SEM images of Al5@NDC (a), (b) with scale bar given in 100 nm and 500 nm, respectively, composite with AuNPs in analogical scales (c), (d), Al4@NDC with a scale bar shown in 200 nm and 500 nm scale (e), (f), Al4Au@NDC with a scale bar 100 nm and 500 nm (g), (h), respectively.

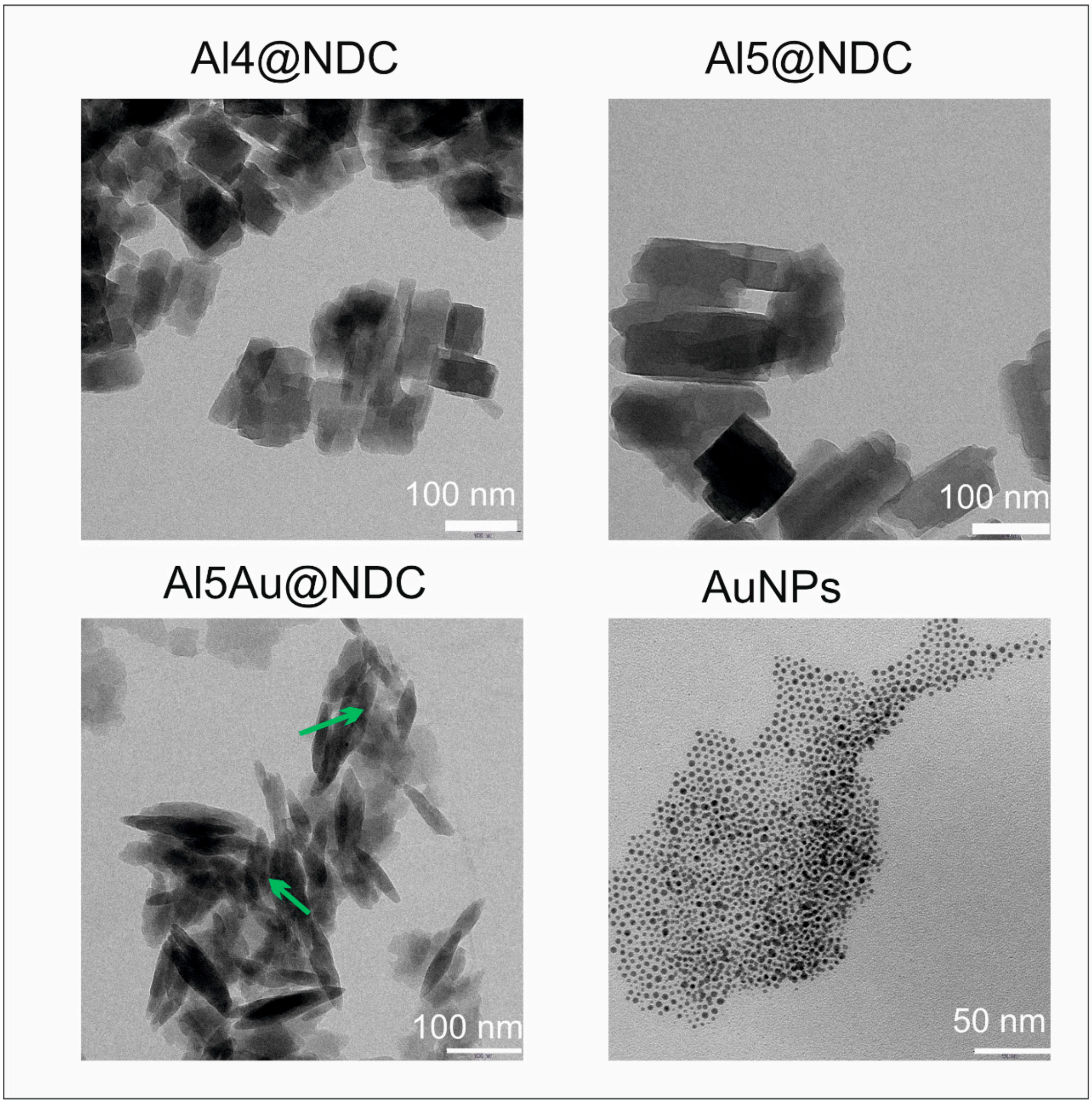

Figure S4. The selected TEM images of the fabricated nanomaterials. The green arrows show AuNPs located inside composite nanocrystals.

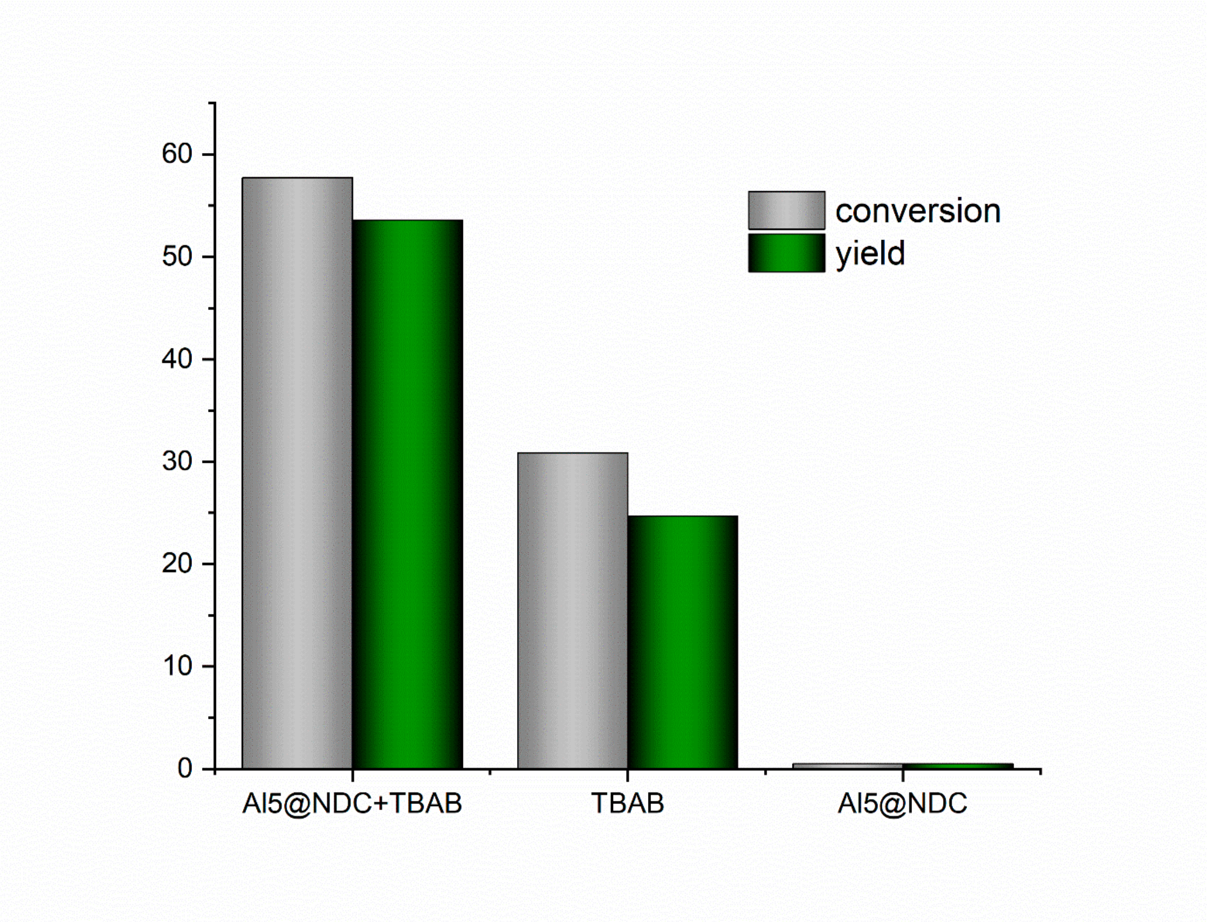

Figure S5. Influence of the fabricated catalyst (Al5@NDC) and cocatalyst (TBAB) separately and together on the cycloaddition of CO_2 to glycidol leading to glycerol carbonate (time of reaction 1 h, t=90°C, 1 MPa CO_2).

Additional information on Powder X-ray diffraction (PXRD)

Powder X-ray diffraction (PXRD) patterns of the MOFs indicated a high degree of crystallinity despite the fine microstructure revealed by the SEM (Figure 1 and Figure 2 in the main text). The data were analyzed using the Expo2014 software[1] to determine the material's most plausible space group and unit cell parameters. MOF Al4 and MOF Al5 lattices, regardless of the presence of gold nanoparticles, conform to the same triclinic P-1 crystallographic system with unit cell parameters listed in Supplementary **Table S1**.

Experimental PXRD patterns and fitted curves are shown in **Figure 8** (main text).

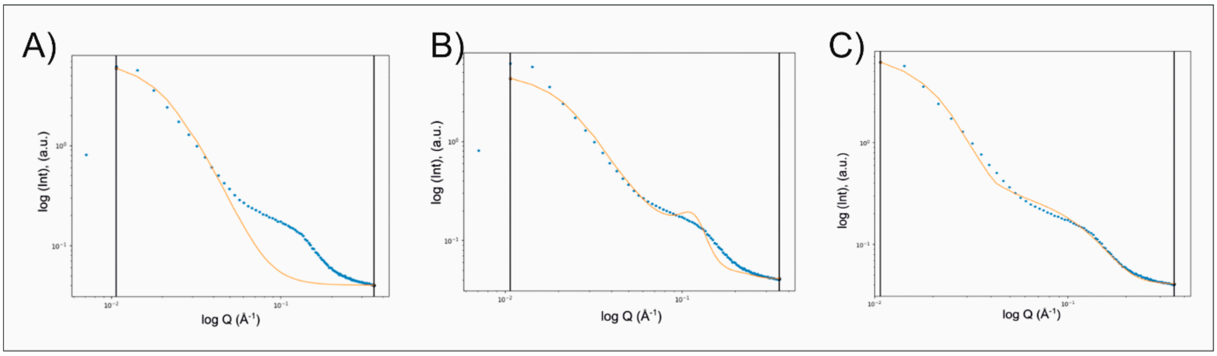

Figure S6. SAXS pattern recorded for AuNPs toluene at 1 wt./vol nanoparticles' and its fitting using:

A) Basic spherical form-factor model where experimental SAXS data (blue points) fitted to spherical non-interacting dilute sphere model (orange curve). Best fit parameters: D_{avg} = 6.2 nm, log-normal dispersity = 0.53.,

B) Hard-sphere structure factor with spherical form-factor model where experimental SAXS data (blue points) fitted to a hard-sphere structure factor model (orange curve). Best fit parameters: D_{avg} = 4.4 nm, log-normal dispersity = 0.61, vol fraction parameter = 0.34.B),

C) Bimodal distribution model Experimental SAXS data (blue points) fitted to a bimodal NPs size distribution model (orange curve). Best fit parameters: D_{sm} = 3.7 nm, D_{lg} = 22.1 nm; volume ratio of small-to-large NPs ≈ 7.7.
Experimental SAXS data (blue points) fitted to a bimodal NPs size distribution model (orange curve) using SasView 5.0.5 (www.sasview.org/) [2].

Table S1

Structural details from PXRD analysis of dried MOF powders

Material	Space group	*a* (Å)	*b* (Å)	*c* (Å)	*alpha* (deg)	*beta* (deg)	*gamma* (deg)
Al4@NDC	P 4/nmm	21.086	21.086	6.926	90	90	90
Al4Au@NDC	P 4/nmm	21.089	21.089	6.453	90	90	90
Al5@NDC	P 4/nmm	21.070	21.070	6.922	90	90	90
Al5Au@NDC	P 4/nmm	21.106	21.106	6.593	90	90	90

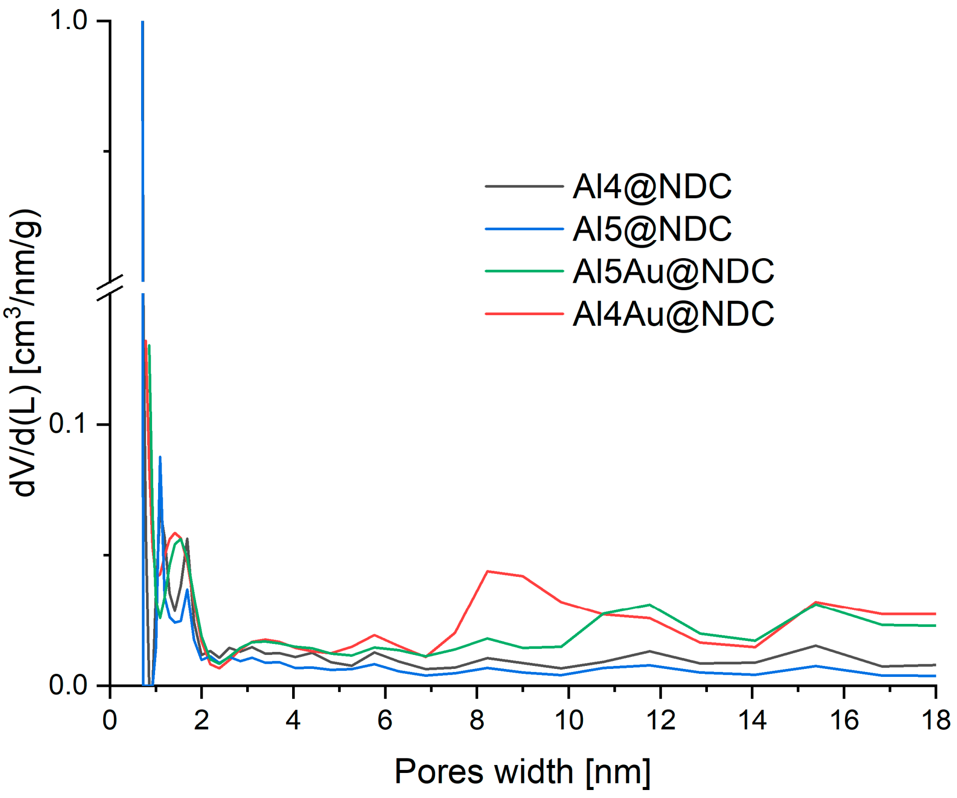

Figure S7. The distribution of pore sizes in the fabricated MOF materials determined from N_2 adsorption analyses.

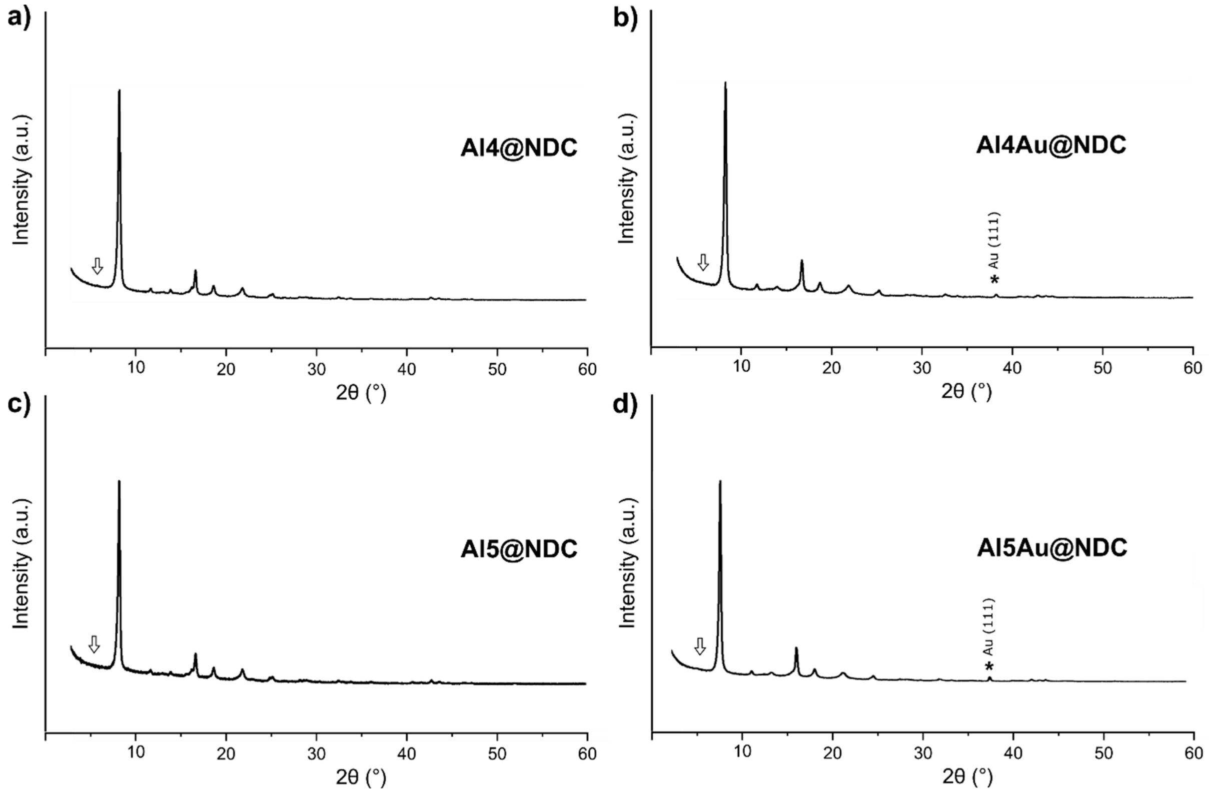

Figure S8. Powder X-ray diffraction (PXRD) patterns of as-synthesized MOF nanomaterials: a) Al4@NDC, b) Al4@NDC, c) Al5@NDC, and d) Al5Au@NDC. The arrows mark the position of the absent reflexes visible in the dried samples. The asterisks in panels b) and d) indicate the (111) reflex originating from gold present in these materials.

References

1. Altomare, A.; Cuocci, C.; Giacovazzo, C.; Moliterni, A.; Rizzi, R.; Corriero, N.; Falcicchio, A., EXPO2013: a kit of tools for phasing crystal structures from powder data. *J. Appl. Crystallogr.* **2013,** 46, (4), 1231-1235.

2. Doucet, M., Cho, J.H., Alina, G., Bakker, J., Bouwman, W., Butler, P., Campbell, K., Gonzales, M., Heenan, R., Jackson, A. and Juhas, P., 2017. SasView Version 4.1. Zenodo and http://www. sasview. org.
